# Anaerobic fixed-target serial crystallography using sandwiched silicon nitride membranes

**DOI:** 10.1107/S205979832300880X

**Published:** 2023-11-01

**Authors:** Monika Bjelčić, Kajsa G. V. Sigfridsson Clauss, Oskar Aurelius, Mirko Milas, Jie Nan, Thomas Ursby

**Affiliations:** a MAX IV Laboratory, Lund University, PO Box 118, SE-221 00 Lund, Sweden; b LINXS Institute of Advanced Neutron and X-ray Science, Lund, Sweden; Fonds National de la Recherche, Luxembourg

**Keywords:** fixed-target serial crystallography, sample supports, sandwiched silicon nitride membranes, anaerobic data collection, hemoglobin

## Abstract

In recent years, the emergence of serial crystallography, initially pioneered at X-ray free-electron lasers (XFELs), has sparked a growing interest in collecting macromolecular crystallographic data at room temperature. Various fixed-target serial crystallography techniques have been developed, ranging from commercially available chips to in-house designs implemented at different synchrotron facilities. Nevertheless, there is currently no commercially available chip (known to the authors) specifically designed for the direct handling of oxygen-sensitive samples. This study presents a methodology employing silicon nitride chips arranged in a ‘sandwich’ configuration, enabling reliable room-temperature data collection from oxygen-sensitive samples. The method involves the utilization of a custom-made 3D-printed assembling tool and a MX sample holder. To validate the effectiveness of the proposed method, deoxyhemoglobin and methemo­globin samples were investigated using the BioMAX X-ray macromolecular crystallo­graphy beamline, the Balder X-ray absorption spectroscopy beamline and UV–Vis absorption spectroscopy.

## Abstract 2

Anaerobic, room-temperature X-ray diffraction and absorption spectroscopy is performed using sandwiched silicon nitride membranes, prepared using 3D-printed tools, in an oxygen-free environment with a reducing agent. Using crystals of hemoglobin as a model system, it was demonstrated that this method ensures oxygen-free data collection.

## 1. Introduction

Serial synchrotron crystallography (SSX) is quickly becoming a popular approach for low-dose, room-temperature structural biology research (Stellato *et al.*, 2014 ▸; Gati *et al.*, 2014 ▸; Coe & Fromme, 2016 ▸; Diederichs & Wang, 2017 ▸). The development of fourth-generation storage-ring light sources (Eriksson *et al.*, 2014 ▸) has enabled X-ray beams with high brilliance and micrometre-scale focusing; combined with advances in fast-frame-rate X-ray detectors (Leonarski *et al.*, 2020 ▸), dedicated SSX beamline endstations are now available for users. Current sample-delivery methods for SSX broadly fall into one of two categories: fixed-target methods, where crystal slurries are deposited onto a substrate that is rastered across the beam (Sherrell *et al.*, 2015 ▸, 2022 ▸; Schulz *et al.*, 2018 ▸; Zielinski *et al.*, 2022 ▸), and flow systems, where crystals suspended in a medium are directly passed through the beam (Botha *et al.*, 2015 ▸; Shilova *et al.*, 2020 ▸; Nam, 2020 ▸; Knoška *et al.*, 2020 ▸; Ghosh *et al.*, 2023 ▸). Still, challenges remain, from the amount of sample used to heating of the system, clogging of capillaries, burning of the material and sample sensitivity. One sample type with specific needs are metalloproteins, which can be estimated to make up a large part of proteomes. Anaerobic sample environments extend the range of redox chemistry that can be characterized by crystallographic means and are of use for studies of redox-active metalloproteins (Handing *et al.*, 2018 ▸). However, the requirement for anaerobicity adds another layer of challenge and different solutions have been proposed, including the use of environmental chambers around the sample position (Fuller *et al.*, 2017 ▸; Mehrabi *et al.*, 2021 ▸) or sealing fixed-target chips under anaerobic conditions (Rabe *et al.*, 2020 ▸).

Hemoglobin (Hb) is an iron-containing heme protein that is essential for oxygen transport. The protein is an assembly of four globular subunits, each containing a heme B (protoporphyrin IX group; Hsia, 1998 ▸). Equine hemoglobin was among the first protein structure to be solved at high resolution (Perutz *et al.*, 1960 ▸). It is a useful model system for oxygen-sensitive metalloproteins due to its ubiquity and its high but reversible affinity for oxygen. Deoxyhemoglobin (DeoxyHb) is missing a sixth ligand in the coordination sphere of the ferrous heme iron (composed of the four heme nitrogens and a histidine) and thus is very sensitive to the presence of oxygen, oxyhemoglobin (OxyHb) has one O_2_ molecule bound to a ferrous heme iron, and methemoglobin (MetHb), the oxidized form of Hb, has a heme iron that is in the +3 (ferric) oxidation state with a water bound in the sixth ligand position (Yang & Phillips, 1996 ▸). Both DeoxyHb and MetHb are used here as proofs of concept for our anaerobic system.

Here, we describe a method for anaerobic SSX at room temperature with silicon nitride membranes that can easily be implemented on another beamline. We have developed a fixed-target method that ‘sandwiches’ oxygen-sensitive crystals in between silicon nitride (SiN) chips (Coquelle *et al.*, 2015 ▸; Shilova *et al.*, 2020 ▸), protecting them from ambient oxygen. Using crystals of DeoxyHb, we demonstrate the practicality of this design for anaerobic SSX, and further verify using UV–Vis and X-ray spectroscopy that the hemoglobin crystals remain in the deoxy state.

## 2. Materials and methods

### 2.1. Preparing microcrystals

Equine hemoglobin (Sigma, CAS 9047-09-0) stock solution at 20–25 mg ml^−1^ was prepared in 10 m*M* HEPES pH 7.5 and crystallized using the stirred batch method (Sato-Tomita & Shibayama, 2017 ▸) by mixing in precipitant solution [26%(*v*/*v*) PEG 3350, 10 m*M* HEPES pH 7.5]. At 20°C, 500 µl precipitant was added to 250 µl protein solution. The mixture was stirred in small HPLC vials with rice-grain-sized stir bars on a magnetic stirrer for about 20 h, after which crystals of 5 × 5 × 5 µm in size were obtained.

### 2.2. 3D-printed holder and accessories

**Figure 1:**
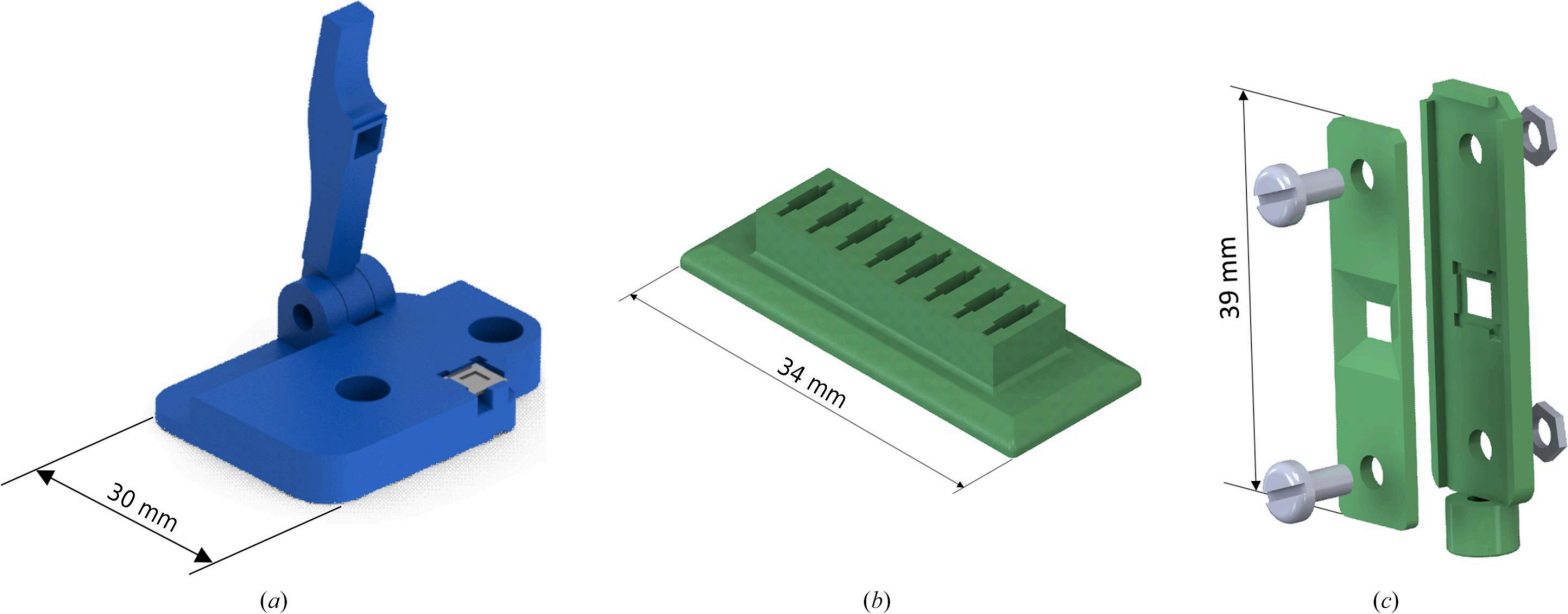
3D-printed accessories for SiN chip handling. (*a*) Assembling tool; (*b*) SiN chip tray; (*c*) MX sample holder.

Since handling this type of chip is challenging due to its small size and thinness, we have designed an assembling tool, a chip tray and a MX sample holder for easier manipulation. The MX sample holder was printed with Tough 1500 Resin (Formlabs, USA), while for the rest of the 3D-printed components Tough Resin V5 (Formlabs, USA) was used. The assembling tool went through a few iterations, as can be seen in Supplementary Fig. S1. The final iteration accounts for imperfections in chip size (some sides being longer or with ‘horns’ in the edges), avoids too high pressure with the lever that would destroy the chips and leaves one corner easily accessible for handling upon assembly (Fig. 1 ▸ *a*). Using the chip tray (Fig. 1 ▸ *b*), several chips can be prepared for quicker assembly. To ease sample mounting to the goniometer head at MX beamlines, the MX sample holder was designed to be easily attached to SPINE bases (Cipriani *et al.*, 2006 ▸; Fig. 1 ▸ *c*). The tightening screws allow extra pressure to be applied to the sides of the sandwiched chips during data collection, further ensuring airtightness.

### 2.3. Anaerobic chip assembly for X-ray crystallography

**Figure 2:**
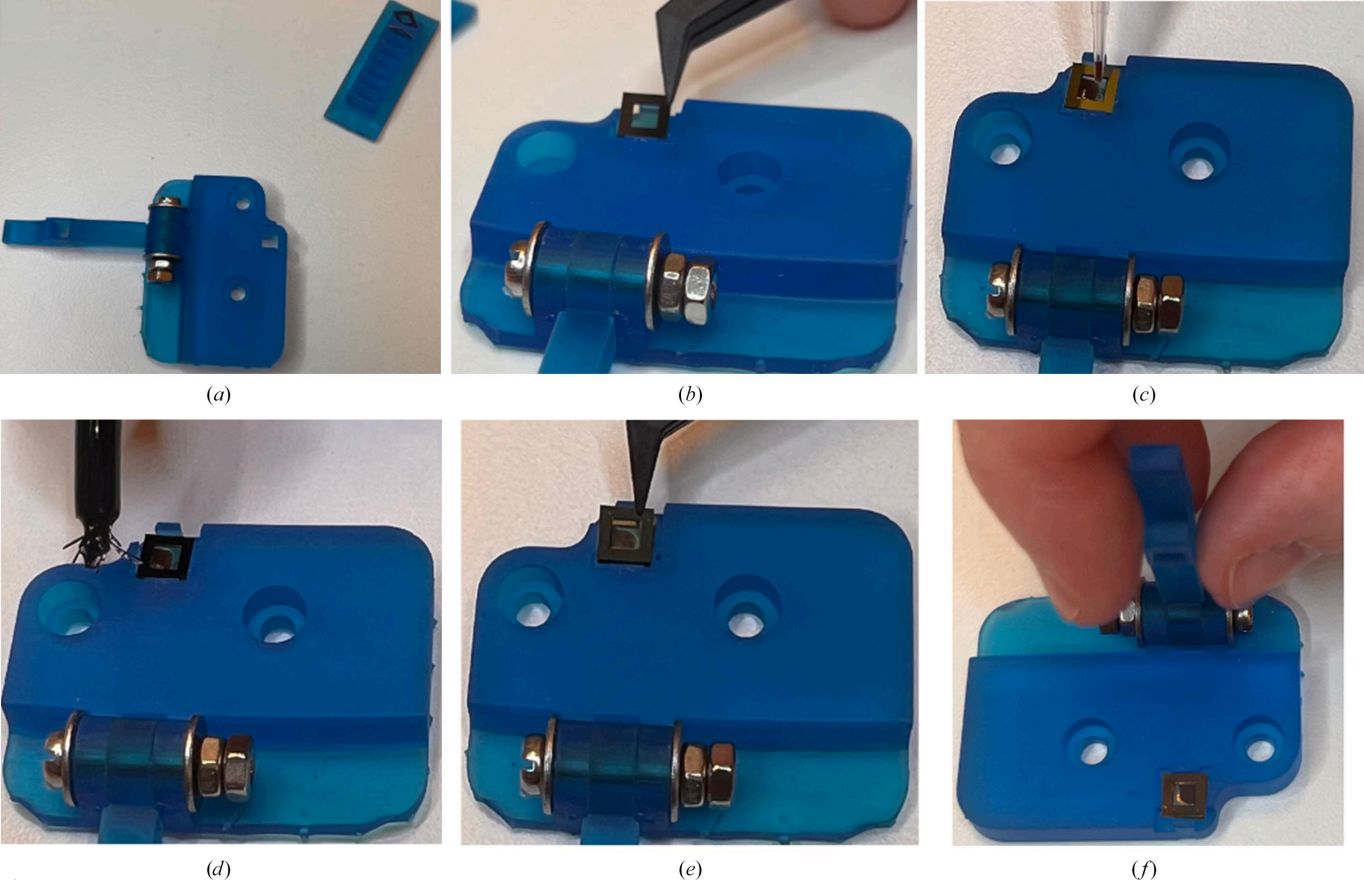
Procedure for anaerobic chip assembly. (*a*) Assembling tool with chip tray. (*b*) The first chip is loaded into the assembling tool. (*c*) 2 µl of sample slurry is gently loaded onto the chip. (*d*) Small drops of superglue are added to all four corners of the chip. (*e*) A second chip is placed on top to create a sealed chip sandwich. (*f*) Chips are gently pressed with the lever of the assembling tool to secure the seal between them.

Before data collection, DeoxyHb crystals were freshly prepared inside a nitrogen-filled glovebox (Coy Laboratory Products). The average oxygen concentration in the glovebox was 2 p.p.m. during sample preparation and never went above 10 p.p.m.. The aerobically grown hemoglobin microcrystals were transferred into the glovebox and chemically reduced by mixing sodium dithionite solution [50 m*M* sodium dithionate, 26%(*w*/*v*) PEG 3350, 10 m*M* HEPES pH 7.5] (Vojtěchovský *et al.*, 1999 ▸) with hemoglobin microcrystals (5 and 20 µl, respectively) and incubated within the glovebox at room temperature for 2 h (during which the crystal slurry undergoes a colour change from brown to pink). Inside the glovebox, SiN chips (membrane size 2.5 × 2.5 mm, membrane thickness 1000 nm, frame size 5.0 × 5.0 mm, frame thickness 200 µm; Silson, United Kingdom) were loaded onto the assembling tool (Figs. 2 ▸ *a* and 2 ▸ *b*), followed by the addition of 2 µl microcrystal slurry (Fig. 2 ▸ *c*). To seal the chip, a small amount of superglue (Loctite Super Glue Brush On) was applied onto all four corners of the chip frame in an L-shape with a single hair from a small paintbrush (Fig. 2 ▸ *d*). A second chip was then placed on the top (Fig. 2 ▸ *e*) and pressed gently with the lever (Fig. 2 ▸ *f*) to secure it and to create a seal, as confirmed by a tiny amount of glue overflowing along all edges. The assembled chip was then placed in the MX sample holder (Fig. 1 ▸ *c*) for data collection.

### 2.4. Anaerobic chip assembly for UV–Vis and X-ray absorption near-edge structure (XANES)

The purpose of the XANES experiment was to establish a time stamp to determine how long the chips can retain the deoxy­hemoglobin form. A special holder was designed to fit six chip sandwiches into the beamline sample environment setup (Supplementary Figs. S2*b* and S2*c*). The chips were assembled in the same way as described in Section 2.3.

UV–Vis spectroscopy using the MX sample holder, with the chip prepared in the exact same way as for X-ray crystallo­graphic data collection, was carried out with an Agilent Cary 60 UV–Vis spectrophotometer in the wavelength range 300–800 nm and followed over 2 h. The UV–Vis spectrophoto­meter was modified with an external optical cable with a probe leading to a black box with a holder for the MX sample holder.

### 2.5. Data collection

#### 2.5.1. Crystallographic data collection

Data for MetHb and DeoxyHb were collected on the BioMAX beamline (Ursby *et al.*, 2020 ▸). The prepared chip sandwich (Supplementary Fig. S2*a*) was mounted and aligned on the gonio­meter. Sample viewing, alignment and measurement were performed using the *MXCuBE*3 beamline-control software (Mueller *et al.*, 2017 ▸). The chip was rastered through the X-ray beam in a regular snake-like pattern defined by a mesh grid drawn on the chip in *MXCuBE*3. The fast shutter was open during each row of the mesh. Rotation of the goniometer was blocked during data collection. Diffraction data were collected at room temperature with an X-ray beam size of 20 × 5 µm (full-width at half-maximum), through a 10 µm diameter aperture, at a photon energy of 12.6 keV and a flux of 2 × 10^12^ photons s^−1^. 10 000–20 000 images per chip were collected from five chips for both MetHb and DeoxyHb. The crystallographic data-acquisition process was split into several smaller mesh scans spaced apart. Consequently, instead of collecting 10 000–20 000 images on a single mesh, they were obtained through several smaller mesh scans. Diffraction data were recorded with an EIGER 16M hybrid pixel detector with an exposure time of 0.011 s. Each crystal received an average radiation dose of ∼40 kGy as calculated using *RADDOSE-3D* (Zeldin *et al.*, 2013 ▸). For the DeoxyHb samples, it took 5–10 min to take the sample from the glovebox, put it inside the beamline hutch and perform data collection.

#### 2.5.2. XANES data collection

XANES data were collected in fluorescence mode on the Balder beamline at MAX IV Laboratory using a silicon drift detector (SDD; X-PIPS 7-element SDD, Mirion Technologies, USA). The liquid-nitrogen-cooled double-crystal (Si111) monochromator (FMB Oxford, United Kingdom) was calibrated to 7112 eV for the first inflexion point of an iron foil for Fe *K*-edge data collection. XANES spectra of the Fe *K* edge were recorded as continuous-fly scans (settings: acquisition time per point 10 ms, energy step 0.2 eV, range 7062–7212 eV, 23 s per scan) to investigate the oxidation state of the heme iron of hemoglobin in the SiN chips. The first chip was used to find and focus the beam; after this, every chip was collected once (each collection consists of five scans performed in direct sequence) with a time separation of 20 min between the chips. In addition to the chips with DeoxyHb crystals, a fully oxygenated crystal sample (a DeoxyHb sample exposed to air for 3 min) was measured. The best measurement strategy was a defocused beam (0.2 × 2 mm) with a manganese filter (*Z* − 1) in front of the fluorescence detector. The incoming flux at the sample was 3 × 10^12^ photons s^−1^. With the given flux and beam parameters, the dose was estimated to be around 67 kGy per single scan (23 s). To obtain the normalized XANES spectrum presented in the figures, fluorescence counts were divided by ten (ionization chamber reading incoming flux) and normalized to unity edge step.

### 2.6. Data processing, model building and refinement

Diffraction data were indexed, integrated, merged and converted to MTZ format using *CrystFEL* 0.10.1 (White, 2019 ▸; White *et al.*, 2012 ▸). The indexing rates were 43.3% and 53.6% for MetHb and DeoxyHb, respectively. Data truncation, phasing and structure refinement were performed in *CCP*4 Cloud (Krissinel *et al.*, 2022 ▸; Agirre *et al.*, 2023 ▸). High-resolution structures of DeoxyHb (PDB entry 1ibe; Wilson *et al.*, 1996 ▸) and MetHb (PDB entry 6sva; Mikolajek *et al.*, 2023 ▸) were used as models for molecular replacement with *Phaser* (McCoy *et al.*, 2007 ▸). The structures were refined by one round of rigid-body refinement using *REFMAC*5 (Nicholls *et al.*, 2018 ▸; Murshudov *et al.*, 1997 ▸, 2011 ▸) followed by several rounds of restrained refinement. Model building was performed in *Coot* (Emsley *et al.*, 2010 ▸; Krissinel & Henrick, 2004 ▸) and all structural representations were generated in *PyMOL* (Schrödinger). Room-temperature SSX structures of MetHb and DeoxyHb were obtained at 1.95 and 1.85 Å resolution, respectively. Data-collection and refinement statistics are presented in Table 1 ▸. The DeoxyHb chips were also processed and refined separately to investigate them individually (data-collection and refinement statistics are presented in Supplementary Table S1).

## 3. Results

### 3.1. Structural differences between deoxy and met forms

**Figure 3:**
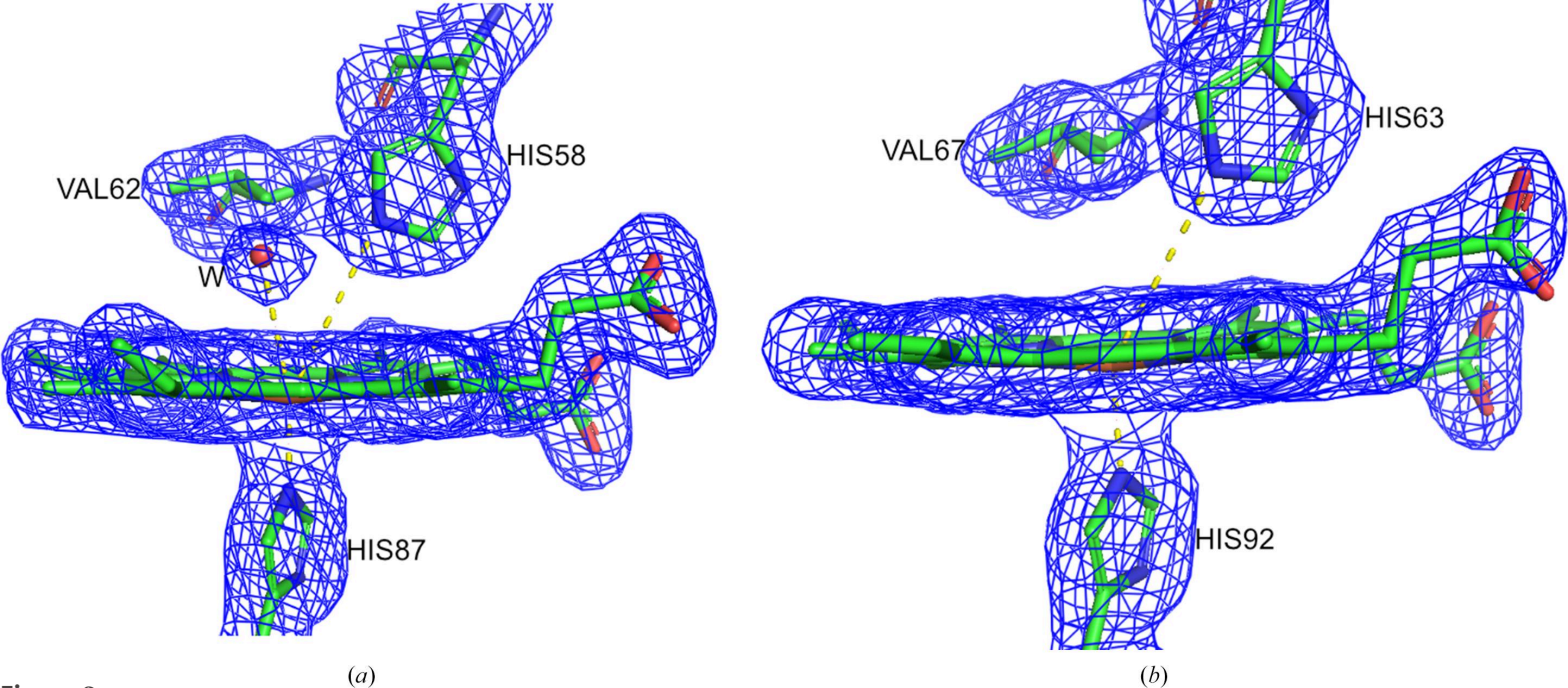
View of the final 2*mF*_o_ − *DF*_c_ electron-density map for the heme pockets of DeoxyHb: (*a*) the α subunit and (*b*) the β subunit. Density is contoured at 1.1σ. The distance between His58/63 and the heme iron is 4.2 Å, while the distance between the water molecule and the heme iron is 3.1 Å

**Figure 4:**
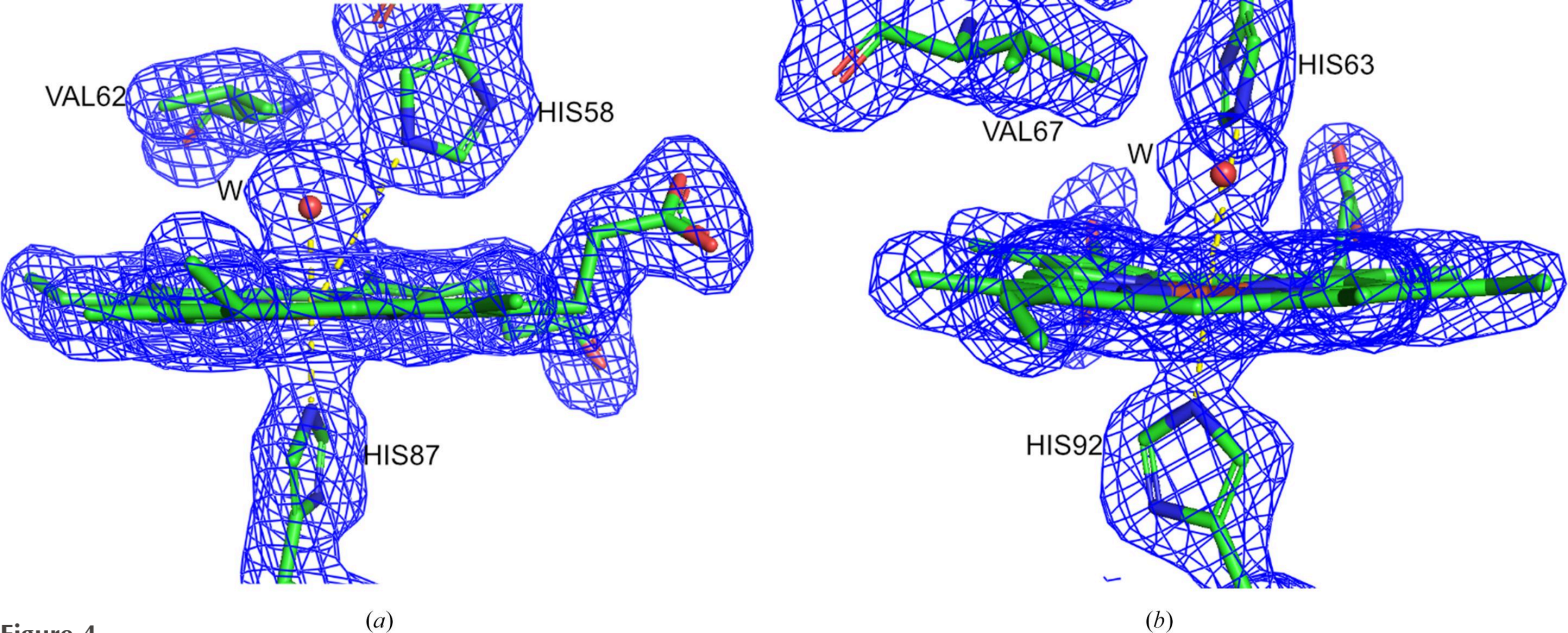
View of the final 2*mF*_o_ − *DF*_c_ electron-density map for the heme pockets of MetHb: (*a*) the α subunit and (*b*) the β subunit. Density is contoured at 1.1σ. The distance between His58/63 and the heme iron is 4.1 Å, while the distance between the water molecule and the heme iron is 2.1 Å

The α-subunits of DeoxyHb have a water molecule in the heme pocket about 3 Å (3.1 Å in this case) from the iron and hydrogen-bonded to the distal histidine (Fig. 3 ▸). In MetHb the α- and β-heme groups have a water molecule as the sixth ligand, approximately 2 Å (2.2 Å in this case) from the iron (Fig. 4 ▸). When hemoglobin changes from the deoxy form to the oxy or met form there is no significant change in the inner region of the α1β1/α2β2 dimer. Visible changes are present in the outer region of the dimer and in the dimer-interface area. The interaction between α1β2 and α2β1 moves towards the centre of the molecule. Residues in contact in the αβ interfaces are between αB (Glu30/A–Phe36/A) and βH (Phe122/B–Lys132/B), αG (Ser102/A–Leu113/A) and βG (Gly107/B–Phe118/B), and αH (Phe117/A–Lys127/A) and βB (Arg30/B–Pro36/B). The most noticeable movement is at the β-heme, where a water molecule is bound in MetHb compared with the empty β-heme in DeoxyHb. The β-heme of MetHb moves downwards to the proximal side of the heme pocket (Supplementary Fig. S3). Both structures are in agreement with published structures (Baldwin & Chothia, 1979 ▸; Liddington *et al.*, 1992 ▸; Wilson *et al.*, 1996 ▸).

All five chips with DeoxyHb that were also independently processed exhibited the presence of the deoxy state (Supplementary Table S1 and Supplementary Figs. S12–S15), with the exception of chip 3 that contained a bubble following the preparation, resulting in too few indexed images for a separate analysis.

### 3.2. XANES data

**Figure 5:**
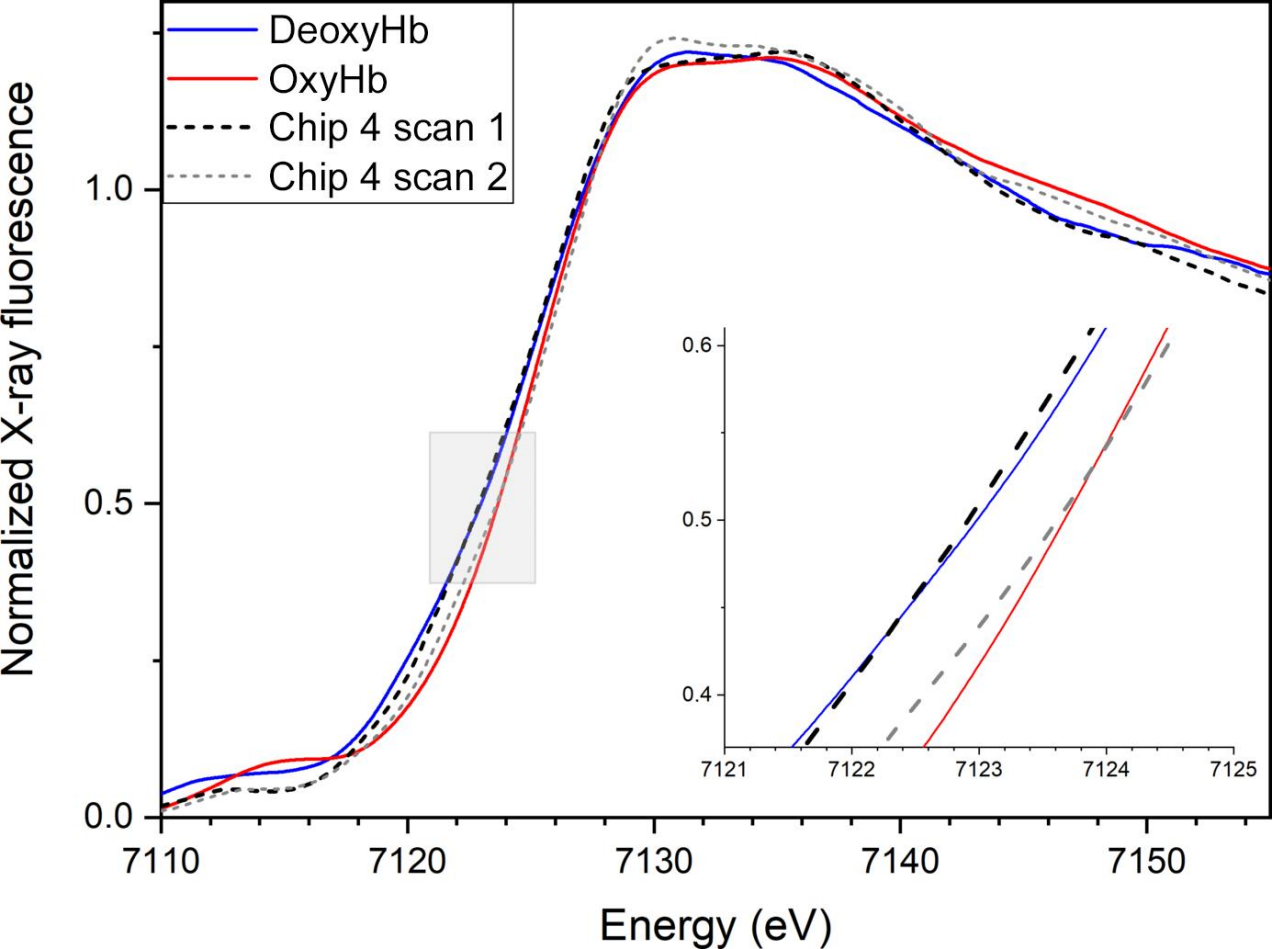
The Fe *K*-edge XANES of hemoglobin crystals in the deoxy state (blue, single scan) and in the fully oxygenated state (red) compared with crystals in SiN chip 4: first scan (black dashed line) and second scan (grey dashed line). Inset, a close-up view of the edge position from the area marked with a grey rectangle in the main figure.

From the preliminary test of the chip sandwiches with an X-ray beam at the Balder beamline, we learned that the Fe *K* edge shifts after long exposure (60 s). The position of the Fe *K* edge shifted to higher energy after the first scan (30 s), and by the second XANES scan the edge had already approached the position for fully oxygenated hemoglobin. This fact was used in a strategy to find out how long the chip seal lasts under ambient conditions in air by waiting 20 min between starting to scan each of the consecutive chip sandwiches, *i.e.* only the first scan of each chip was compared (10 s to reach the edge, 23 s full scan). XANES data for the six individual chip sandwiches mounted in the chip holder made for the Balder beamline showed that two of the chip sandwiches (chips 3 and 4) had intact seals when the measurement started (*i.e.* were still in the deoxy state; Supplementary Fig. S4), as their absorption edges were not shifted to higher energy as observed on O_2_ binding. This gave us a time stamp for the seal of at least 40 min (the time between the start of scan 1 of chip 2 and of chip 4). Fig. 5 ▸ shows a comparison of the edge position of chip 4 upon the first scan and the second scan with the deoxy and oxy states, respectively. The conclusion from this comparison is that only the first scan of chip 4 (black dashed line) had an edge position corresponding to the hemoglobin deoxy state (blue line), while the second scan (grey dashed line) had already shifted to the position of the oxygenated state (red line).

### 3.3. UV–Vis data

**Figure 6:**
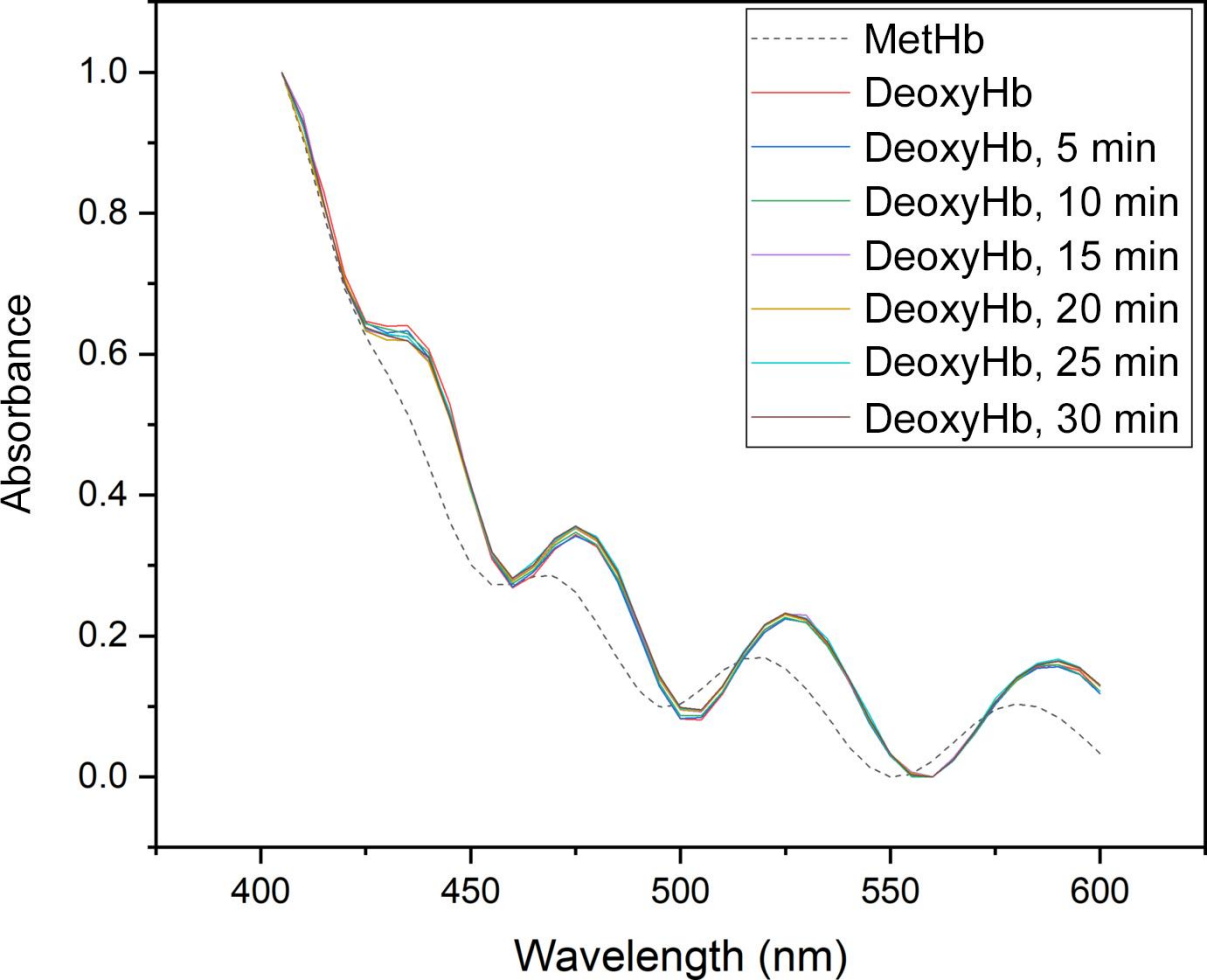
UV–Vis spectra of a chip sandwich (chip 1) with DeoxyHb in the MX sample holder (the spectra have been normalized in *Origin* 2018b).

Chips were prepared as described in Section 2.3. The samples in both chips measured stayed pink, and neither chip changed from the deoxy to the met form in the first 30 min according to the spectra (Fig. 6 ▸ and Supplementary Fig. S11). The second chip was also measured 1 h 30 min and 2 h 10 min after the start of the experiment (Supplementary Fig. S11). These later time points show a change from the deoxy form, which could also be seen by eye as the sample colour changed from pink to brown.

## 4. Discussion

As demonstrated by a combination of XANES, UV–Vis absorption spectroscopy and X-ray crystallography experiments, the described method slows down reoxidation of the hemoglobin to a level that allows data collection within a reasonable time frame for oxygen-sensitive samples. The XANES data showed that the hemoglobin crystals stayed in the deoxy form for at least 40 min (see Fig. 5 ▸ and Supplementary Fig. S4). The edge-scan data revealed a relatively low success rate of the chip sandwiches in the Balder holder, which was primarily attributed to the design of the multichip holder. Specifically, the middle row positions (chips 3 and 4) experienced pressure from both sides of the SiN chip through a two-screw mechanism, as depicted in Supplementary Fig. S2. Conversely, the upper and lower rows of the holder applied pressure to the chip from only one side (one screw). This asymmetrical pressure distribution on the SiN seals seems to have resulted in leaks (chips 2, 5 and 6) or an increased susceptibility to breakage. Notably, only chips 3 and 4 successfully preserved the deoxy form. In the light of these observations, a new holder for crystallographic collection was developed to maintain an even pressure on all four sides of a single chip, as illustrated in Fig. 1 ▸(*c*). The viability of the newly designed MX sample holder was confirmed using UV–Vis spectroscopic analysis, which demonstrated the sustained retention of the deoxy state over more than 30 min (Fig. 6 ▸ and Supplementary Fig. S11). To establish the consistency of the method, crystallographic data were collected from a total of five chips, and all four chips that gave sufficiently complete data exhibited the presence of the deoxy state (Supplementary Table S1 and Supplementary Figs. S12–S15). Based on the XANES data, it became evident that X-ray exposure causes the iron edge to shift to a higher energy, although it is not clear whether this is because the chip seal breaks or whether it is due to some other effect. The reason for this observed edge shift may be either binding of O_2_ to the ferrous iron (OxyHb) or oxidation of iron to the ferric state (MetHb). The doses of the first XANES scan and of the crystallographic measurements were similar (70 and 40 kGy, respectively), and both showed the presence of the deoxy form. This dose is lower than that recently determined to be a safe dose for SSX experiments (around 380 kGy; de la Mora *et al.*, 2020 ▸), but while this was the case for lysozyme, we have studied a heme protein that may be expected to be more sensitive to radiation damage. It is advisable to keep the dose low and independently verify the oxidation state through spectroscopy if possible, as other samples might have a different sensitivity to X-ray radiation.

The assembling tool for chip-sandwich preparation simplifies the process, facilitates visualization and decreases the risk of crystal or chip damage. CAD models of the tools presented are made available in the supporting information. Furthermore, SiN chips do not cause strong diffraction that can damage detectors, but are single use only. Using the MX sample holder and the protocol presented, it is feasible to collect data from oxygen-sensitive samples and the method can be implemented at many MX beamlines that have access to a glovebox and an MX compatible goniometer.

## Supplementary Material

PDB reference: methemoglobin, 8puq

PDB reference: dexyhemoglobin, 8pur

Data for 3D printing: sssembling tool 1. DOI: 10.1107/S205979832300880X/tz5111sup1.bin

Data for 3D printing: assembling tool 2. DOI: 10.1107/S205979832300880X/tz5111sup2.bin

Data for 3D printing: chip tray. DOI: 10.1107/S205979832300880X/tz5111sup3.bin

Data for 3D printing: hand adjuster. DOI: 10.1107/S205979832300880X/tz5111sup4.bin

Data for 3D printing: MX sample holder 1. DOI: 10.1107/S205979832300880X/tz5111sup5.bin

Data for 3D printing: MX sample holder 2. DOI: 10.1107/S205979832300880X/tz5111sup6.bin

Supplementary Table and Figures. DOI: 10.1107/S205979832300880X/tz5111sup7.pdf

## Figures and Tables

**Table 1:** Data-collection and refinement statistics Values in parentheses are for the highest resolution shell.

	MetHb	DeoxyHb
PDB code	8puq	8pur
Data collection		
Diffraction source	BioMAX, MAX IVLaboratory	BioMAX, MAX IVLaboratory
Wavelength (Å)	0.9763	0.9763
Temperature (K)	294	294
Space group	*C*121	*C*121
*a*, *b*, *c* (Å)	108.29, 63.06, 54.75	108.29, 63.06, 54.75
α, β, γ (°)	90, 111, 90	90, 111, 90
Resolution (Å)	53.55–1.95 (2.02–1.95)	53.50–1.85 (1.91–1.85)
*R*_split_ † (%)	7.50 (41.94)	6.71 (22.28)
〈*I*/σ(*I*)〉	10.60 (1.37)	12.48 (2.90)
CC_1/2_	0.9903 (0.7593)	0.9914 (0.9049)
Completeness (%)	100 (100)	100 (100)
Multiplicity	176 (114)	205 (115)
Collected images	97424	149207
Indexed patterns	42148	80035
Indexing rate (%)	43.3	53.6
No. of reflections	4447049	6070870
No. of unique reflections	25258	29515
Refinement		
Resolution range (Å)	53.49–1.95	53.50–1.85
*R*_work_/*R*_free_ (%)	12.52/17.41	12.99/16.22
No. of atoms	4644	4609
Average *B* factor (Å^2^)	66	74
R.m.s.d., bond lengths (Å)	0.016	0.015
R.m.s.d., angles (°)	1.97	1.99

† *R*_split_ = 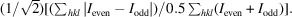

## References

[bb1] Agirre, J., Atanasova, M., Bagdonas, H., Ballard, C. B., Baslé, A., Beilsten-Edmands, J., Borges, R. J., Brown, D. G., Burgos-Mármol, J. J., Berrisford, J. M., Bond, P. S., Caballero, I., Catapano, L., Chojnowski, G., Cook, A. G., Cowtan, K. D., Croll, T. I., Debreczeni, J. É., Devenish, N. E., Dodson, E. J., Drevon, T. R., Emsley, P., Evans, G., Evans, P. R., Fando, M., Foadi, J., Fuentes-Montero, L., Garman, E. F., Gerstel, M., Gildea, R. J., Hatti, K., Hekkelman, M. L., Heuser, P., Hoh, S. W., Hough, M. A., Jenkins, H. T., Jiménez, E., Joosten, R. P., Keegan, R. M., Keep, N., Krissinel, E. B., Kolenko, P., Kovalevskiy, O., Lamzin, V. S., Lawson, D. M., Lebedev, A. A., Leslie, A. G. W., Lohkamp, B., Long, F., Malý, M., McCoy, A. J., McNicholas, S. J., Medina, A., Millán, C., Murray, J. W., Murshudov, G. N., Nicholls, R. A., Noble, M. E. M., Oeffner, R., Pannu, N. S., Parkhurst, J. M., Pearce, N., Pereira, J., Perrakis, A., Powell, H. R., Read, R. J., Rigden, D. J., Rochira, W., Sammito, M., Sánchez Rodríguez, F., Sheldrick, G. M., Shelley, K. L., Simkovic, F., Simpkin, A. J., Skubak, P., Sobolev, E., Steiner, R. A., Stevenson, K., Tews, I., Thomas, J. M. H., Thorn, A., Valls, J. T., Uski, V., Usón, I., Vagin, A., Velankar, S., Vollmar, M., Walden, H., Waterman, D., Wilson, K. S., Winn, M. D., Winter, G., Wojdyr, M. & Yamashita, K. (2023). *Acta Cryst.* D**79**, 449–461.

[bb2] Baldwin, J. & Chothia, C. (1979). *J. Mol. Biol.* **129**, 175–220. 10.1016/0022-2836(79)90277-8 39173

[bb4] Botha, S., Nass, K., Barends, T. R. M., Kabsch, W., Latz, B., Dworkowski, F., Foucar, L., Panepucci, E., Wang, M., Shoeman, R. L., Schlichting, I. & Doak, R. B. (2015). *Acta Cryst.* D**71**, 387–397. 10.1107/S1399004714026327 25664750

[bb6] Cipriani, F., Felisaz, F., Launer, L., Aksoy, J.-S., Caserotto, H., Cusack, S., Dallery, M., di-Chiaro, F., Guijarro, M., Huet, J., Larsen, S., Lentini, M., McCarthy, J., McSweeney, S., Ravelli, R., Renier, M., Taffut, C., Thompson, A., Leonard, G. A. & Walsh, M. A. (2006). *Acta Cryst.* D**62**, 1251–1259. 10.1107/S0907444906030587 17001102

[bb7] Coe, J. & Fromme, P. (2016). *Protein Pept. Lett.* **23**, 255–272. 10.2174/0929866523666160120152937 PMC4868546 26786767

[bb8] Coquelle, N., Brewster, A. S., Kapp, U., Shilova, A., Weinhausen, B., Burghammer, M. & Colletier, J.-P. (2015). *Acta Cryst.* D**71**, 1184–1196. 10.1107/S1399004715004514 PMC4427202 25945583

[bb9] Diederichs, K. & Wang, M. (2017). *Methods Mol. Biol.* **1607**, 239–272. 10.1007/978-1-4939-7000-1_10 28573576

[bb10] Emsley, P., Lohkamp, B., Scott, W. G. & Cowtan, K. (2010). *Acta Cryst.* D**66**, 486–501. 10.1107/S0907444910007493 PMC2852313 20383002

[bb11] Eriksson, M., van der Veen, J. F. & Quitmann, C. (2014). *J. Synchrotron Rad.* **21**, 837–842. 10.1107/S1600577514019286 25177975

[bb12] Fuller, F. D., Gul, S., Chatterjee, R., Burgie, E. S., Young, I. D., Lebrette, H., Srinivas, V., Brewster, A. S., Michels-Clark, T., Clinger, J. A., Andi, B., Ibrahim, M., Pastor, E., de Lichtenberg, C., Hussein, R., Pollock, C. J., Zhang, M., Stan, C. A., Kroll, T., Fransson, T., Weninger, C., Kubin, M., Aller, P., Lassalle, L., Bräuer, P., Miller, M. D., Amin, M., Koroidov, S., Roessler, C. G., Allaire, M., Sierra, R. G., Docker, P. T., Glownia, J. M., Nelson, S., Koglin, J. E., Zhu, D., Chollet, M., Song, S., Lemke, H., Liang, M., Sokaras, D., Alonso-Mori, R., Zouni, A., Messinger, J., Bergmann, U., Boal, A. K., Bollinger, J. M. Jr, Krebs, C., Högbom, M., Phillips, G. N., Vierstra, R. D., Sauter, N. K., Orville, A. M., Kern, J., Yachandra, V. K. & Yano, J. (2017). *Nat. Methods*, **14**, 443–449.

[bb13] Gati, C., Bourenkov, G., Klinge, M., Rehders, D., Stellato, F., Oberthür, D., Yefanov, O., Sommer, B. P., Mogk, S., Duszenko, M., Betzel, C., Schneider, T. R., Chapman, H. N. & Redecke, L. (2014). *IUCrJ*, **1**, 87–94. 10.1107/S2052252513033939 PMC4062088 25075324

[bb14] Ghosh, S., Zorić, D., Dahl, P., Bjelčić, M., Johannesson, J., Sandelin, E., Borjesson, P., Björling, A., Banacore, A., Edlund, P., Aurelius, O., Milas, M., Nan, J., Shilova, A., Gonzalez, A., Mueller, U., Brändén, G. & Neutze, R. (2023). *J. Appl. Cryst.* **56**, 449–460. 10.1107/S1600576723001036 PMC10077854 37032973

[bb15] Handing, K. B., Niedzialkowska, E., Shabalin, I. G., Kuhn, M. L., Zheng, H. & Minor, W. (2018). *Nat. Protoc.* **13**, 1062–1090. 10.1038/nprot.2018.018 PMC6235626 29674755

[bb3] Hsia, C. C. W. (1998). *N. Engl. J. Med.* **338**, 239–248. 10.1056/NEJM199801223380407 9435331

[bb16] Knoška, J., Adriano, L., Awel, S., Beyerlein, K. R., Yefanov, O., Oberthuer, D., Peña Murillo, G. E., Roth, N., Sarrou, I., Villanueva-Perez, P., Wiedorn, M. O., Wilde, F., Bajt, S., Chapman, H. N. & Heymann, M. (2020). *Nat. Commun.* **11**, 657. 10.1038/s41467-020-14434-6 PMC6994545 32005876

[bb17] Krissinel, E. & Henrick, K. (2004). *Acta Cryst.* D**60**, 2256–2268. 10.1107/S0907444904026460 15572779

[bb18] Krissinel, E., Lebedev, A. A., Uski, V., Ballard, C. B., Keegan, R. M., Kovalevskiy, O., Nicholls, R. A., Pannu, N. S., Skubák, P., Berrisford, J., Fando, M., Lohkamp, B., Wojdyr, M., Simpkin, A. J., Thomas, J. M. H., Oliver, C., Vonrhein, C., Chojnowski, G., Basle, A., Purkiss, A., Isupov, M. N., McNicholas, S., Lowe, E., Triviño, J., Cowtan, K., Agirre, J., Rigden, D. J., Uson, I., Lamzin, V., Tews, I., Bricogne, G., Leslie, A. G. W. & Brown, D. G. (2022). *Acta Cryst.* D**78**, 1079–1089.

[bb19] Leonarski, F., Mozzanica, A., Brückner, M., Lopez-Cuenca, C., Redford, S., Sala, L., Babic, A., Billich, H., Bunk, O., Schmitt, B. & Wang, M. (2020). *Struct. Dyn.* **7**, 014305. 10.1063/1.5143480 PMC7044001 32128347

[bb20] Liddington, R., Derewenda, Z., Dodson, E., Hubbard, R. & Dodson, G. (1992). *J. Mol. Biol.* **228**, 551–579. 10.1016/0022-2836(92)90842-8 1453464

[bb21] McCoy, A. J., Grosse-Kunstleve, R. W., Adams, P. D., Winn, M. D., Storoni, L. C. & Read, R. J. (2007). *J. Appl. Cryst.* **40**, 658–674. 10.1107/S0021889807021206 PMC2483472 19461840

[bb22] Mehrabi, P., von Stetten, D., Leimkohl, J.-P., Tellkamp, F. & Schulz, E. C. (2021). *bioRxiv*, 2021.11.07.467596.

[bb5] Mikolajek, H., Sanchez-Weatherby, J., Sandy, J., Gildea, R. J., Campeotto, I., Cheruvara, H., Clarke, J. D., Foster, T., Fujii, S., Paulsen, I. T., Shah, B. S. & Hough, M. A. (2023). *IUCrJ*, **10**, 420–429. 10.1107/S2052252523003810 PMC10324489 37199504

[bb23] Mora, E. de la, Coquelle, N., Bury, C. S., Rosenthal, M., Holton, J. M., Carmichael, I., Garman, E. F., Burghammer, M., Colletier, J.-P. & Weik, M. (2020). *Proc. Natl Acad. Sci. USA*, **117**, 4142–4151. 10.1073/pnas.1821522117 PMC7049125 32047034

[bb24] Mueller, U., Thunnissen, M., Nan, J., Eguiraun, M., Bolmsten, F., Milàn-Otero, A., Guijarro, M., Oscarsson, M., de Sanctis, D. & Leonard, G. (2017). *Synchrotron Radiat. News.* **30**, 22–27.

[bb25] Murshudov, G. N., Skubák, P., Lebedev, A. A., Pannu, N. S., Steiner, R. A., Nicholls, R. A., Winn, M. D., Long, F. & Vagin, A. A. (2011). *Acta Cryst.* D**67**, 355–367. 10.1107/S0907444911001314 PMC3069751 21460454

[bb26] Murshudov, G. N., Vagin, A. A. & Dodson, E. J. (1997). *Acta Cryst.* D**53**, 240–255. 10.1107/S0907444996012255 15299926

[bb27] Nam, K. H. (2020). *J. Appl. Cryst.* **53**, 45–50.

[bb28] Nicholls, R. A., Tykac, M., Kovalevskiy, O. & Murshudov, G. N. (2018). *Acta Cryst.* D**74**, 492–505. 10.1107/S2059798318007313 PMC6096485 29872001

[bb29] Perutz, M., Rossmann, M., Cullis, A. F., Muirhead, H., Will, G. & North, A. C. T. (1960). *Nature*, **185**, 416–422. 10.1038/185416a0 18990801

[bb30] Rabe, P., Beale, J. H., Butryn, A., Aller, P., Dirr, A., Lang, P. A., Axford, D. N., Carr, S. B., Leissing, T. M., McDonough, M. A., Davy, B., Ebrahim, A., Orlans, J., Storm, S. L. S., Orville, A. M., Schofield, C. J. & Owen, R. L. (2020). *IUCrJ*, **7**, 901–912. 10.1107/S2052252520010374 PMC7467159 32939282

[bb31] Sato-Tomita, A. & Shibayama, N. (2017). *Crystals*, **7**, 282.

[bb33] Schulz, E. C., Mehrabi, P., Müller-Werkmeister, H. M., Tellkamp, F., Jha, A., Stuart, W., Persch, E., De Gasparo, R., Diederich, F., Pai, E. F. & Miller, R. J. D. (2018). *Nat. Methods*, **15**, 901–904. 10.1038/s41592-018-0180-2 30377366

[bb34] Sherrell, D. A., Foster, A. J., Hudson, L., Nutter, B., O’Hea, J., Nelson, S., Paré-Labrosse, O., Oghbaey, S., Miller, R. J. D. & Owen, R. L. (2015). *J. Synchrotron Rad.* **22**, 1372–1378. 10.1107/S1600577515016938 PMC4629865 26524301

[bb35] Sherrell, D. A., Lavens, A., Wilamowski, M., Kim, Y., Chard, R., Lazarski, K., Rosenbaum, G., Vescovi, R., Johnson, J. L., Akins, C., Chang, C., Michalska, K., Babnigg, G., Foster, I. & Joachimiak, A. (2022). *J. Synchrotron Rad.* **29**, 1141–1151. 10.1107/S1600577522007895 PMC9455217 36073872

[bb36] Shilova, A., Lebrette, H., Aurelius, O., Nan, J., Welin, M., Kovacic, R., Ghosh, S., Safari, C., Friel, R. J., Milas, M., Matej, Z., Högbom, M., Brändén, G., Kloos, M., Shoeman, R. L., Doak, B., Ursby, T., Håkansson, M., Logan, D. T. & Mueller, U. (2020). *J. Synchrotron Rad.* **27**, 1095–1102. 10.1107/S1600577520008735 PMC7467353 32876583

[bb37] Stellato, F., Oberthür, D., Liang, M., Bean, R., Gati, C., Yefanov, O., Barty, A., Burkhardt, A., Fischer, P., Galli, L., Kirian, R. A., Meyer, J., Panneerselvam, S., Yoon, C. H., Chervinskii, F., Speller, E., White, T. A., Betzel, C., Meents, A. & Chapman, H. N. (2014). *IUCrJ*, **1**, 204–212. 10.1107/S2052252514010070 PMC4107920 25075341

[bb38] Ursby, T., Åhnberg, K., Appio, R., Aurelius, O., Barczyk, A., Bartalesi, A., Bjelčić, M., Bolmsten, F., Cerenius, Y., Doak, R. B., Eguiraun, M., Eriksson, T., Friel, R. J., Gorgisyan, I., Gross, A., Haghighat, V., Hennies, F., Jagudin, E., Norsk Jensen, B., Jeppsson, T., Kloos, M., Lidon-Simon, J., de Lima, G. M. A., Lizatovic, R., Lundin, M., Milan-Otero, A., Milas, M., Nan, J., Nardella, A., Rosborg, A., Shilova, A., Shoeman, R. L., Siewert, F., Sondhauss, P., Talibov, V. O., Tarawneh, H., Thånell, J., Thunnissen, M., Unge, J., Ward, C., Gonzalez, A. & Mueller, U. (2020). *J. Synchrotron Rad.* **27**, 1415–1429. 10.1107/S1600577520008723 PMC7467343 32876619

[bb39] Vojtěchovský, J., Chu, K., Berendzen, J., Sweet, R. M. & Schlichting, I. (1999). *Biophys. J.* **77**, 2153–2174. 10.1016/S0006-3495(99)77056-6 PMC1300496 10512835

[bb40] White, T. A. (2019). *Acta Cryst.* D**75**, 219–233. 10.1107/S205979831801238X PMC6400257 30821710

[bb41] White, T. A., Kirian, R. A., Martin, A. V., Aquila, A., Nass, K., Barty, A. & Chapman, H. N. (2012). *J. Appl. Cryst.* **45**, 335–341.

[bb42] Wilson, J., Phillips, K. & Luisi, B. (1996). *J. Mol. Biol.* **264**, 743–756. 10.1006/jmbi.1996.0674 8980683

[bb43] Yang, F. & Phillips, G. N. (1996). *J. Mol. Biol.* **256**, 762–774. 10.1006/jmbi.1996.0123 8642596

[bb44] Zeldin, O. B., Gerstel, M. & Garman, E. F. (2013). *J. Appl. Cryst.* **46**, 1225–1230.

[bb45] Zielinski, K. A., Prester, A., Andaleeb, H., Bui, S., Yefanov, O., Catapano, L., Henkel, A., Wiedorn, M. O., Lorbeer, O., Crosas, E., Meyer, J., Mariani, V., Domaracky, M., White, T. A., Fleckenstein, H., Sarrou, I., Werner, N., Betzel, C., Rohde, H., Aepfelbacher, M., Chapman, H. N., Perbandt, M., Steiner, R. A. & Oberthuer, D. (2022). *IUCrJ*, **9**, 778–791. 10.1107/S2052252522010193 PMC9634612 36381150

