# Supplementary material for: Anaerobic fixed-target serial crystallography using sandwiched silicon nitride membranes
Source: Acta Crystallogr D Struct Biol. 2023 Nov 1;79(Pt 11):1018–25. doi: 10.1107/S205979832300880X (PMC10619425; doi:10.1107/S205979832300880X)
Supplement: Supplementary file 7 [file d-79-01018-sup7.pdf]

STRUCTURAL
BIOLOGY

Volume 79 (2023)

Supporting information for article:

Anaerobic fixed-target serial crystallography using sandwiched silicon nitride membranes

Monika Bjelčić, Kajsa G. V. Sigfridsson Clauss, Oskar Aurelius, Mirko Milas, Jie Nan and Thomas Ursby

Table S1 Data collection and refinement statistics of DeoxyHb collected chips.

	Chip1	Chip2	Chip4	Chip5
Diffraction source	BioMAX, MAX IV Laboratory	BioMAX, MAX IV Laboratory	BioMAX, MAX IV Laboratory	BioMAX, MAX IV Laboratory
Wavelength (Å)	0.9763	0.9763	0.9763	0.9763
Temperature (K)	294	294	294	294
Space group	C121	C121	C121	C121
a, b, c (Å)	108.29, 63.06, 54.75	108.29, 63.06, 54.75	108.29, 63.06, 54.75	108.29, 63.06, 54.75
α, β, γ (°)	90, 111. 90	90, 111. 90	90, 111. 90	90, 111. 90
Resolution (Å)‡	53.5 – 1.95 (2.02 – 1.95)	53.5 – 1.95 (2.02 – 1.95)	53.5 – 1.95 (2.02 – 1.95)	53.5 – 1.95 (2.02 – 1.95)
Rsplit (%)†‡	10.79 (44.24)	13.35 (43.62)	13.17 (39)	12.56 (51.93)
I/σ (I)‡	8.38 (1.81)	7.55 (1.95)	7.67 (2.11)	7.25 (1.6)
$CC_{1/2}$‡	0.9813 (0.7533)	0.9649 (0.7017)	0.9702 (0.7844)	0.9755 (0.6720)
Completeness (%)	100 (100)	100 (100)	100 (100)	100 (100)
Multiplicity‡	65 (38)	50 (30)	50 (29)	52 (31)
Collected images	35955	26176	35722	25529
Indexed patterns	21349	17405	19789	16923
Indexing rate (%)	59.4	66.5	55.4	66.3
Number of reflections	1648462	1270057	1267242	1312501
Number of unique reflections	25215	25215	25215	25215
Refinement				
Resolution range (Å)	53.5 – 1.95	53.5 – 1.95	53.5 – 1.95	53.5 – 1.95
R_{work}/R_{free} (%)	13.24/17.67	13.64/18.68	13.68/18.45	14.04/18.89
Number of atoms	4636	4630	4630	4611
Average B factor (Å^2)	65	63	63	60
RMSD bonds (Å)	0.014	0.013	0.014	0.014
RMSD angles (°)	1.93	1.85	1.85	1.93

† $R_{split} = \left(\frac{1}{\sqrt{2}}\right) \frac{\sum_{hkl} |I_{even} - I_{odd}|}{0.5 \sum_{hkl} (I_{even} + I_{odd})}$

‡ Values in parenthesis correspond to those of the highest-resolution shell.

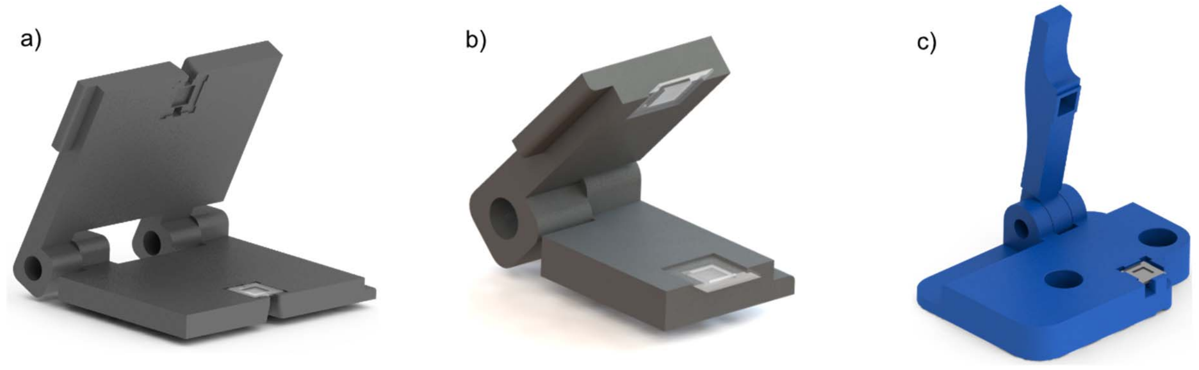

Figure S1 Development of assembling tool, a) first iteration, b) third iteration, c) final iteration.

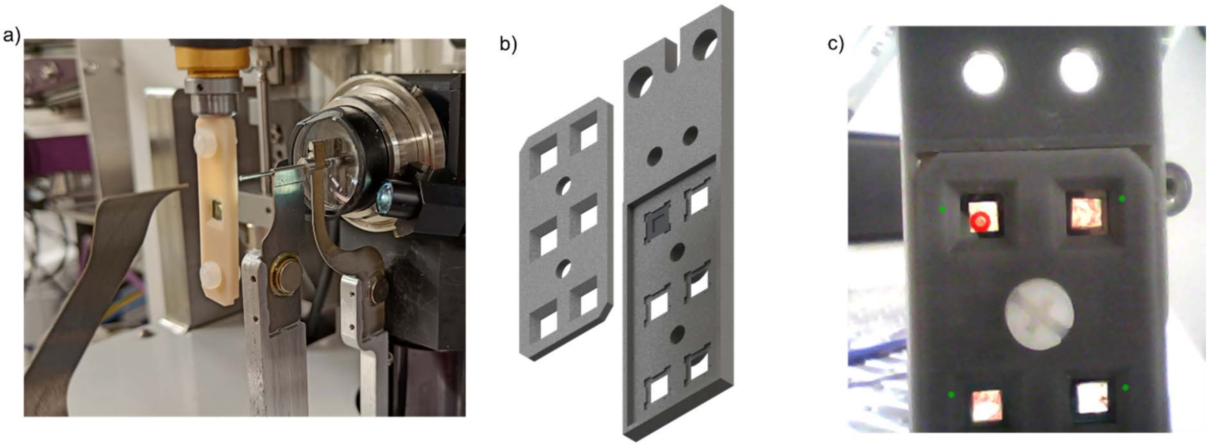

Figure S2 a) MX-sample holder at BioMAX beamline, b) Balder holder, b) Balder holder at the Balder beamline with the red circle marking the beam position on chip 1 seen through the sample camera and Orthoviewer software.

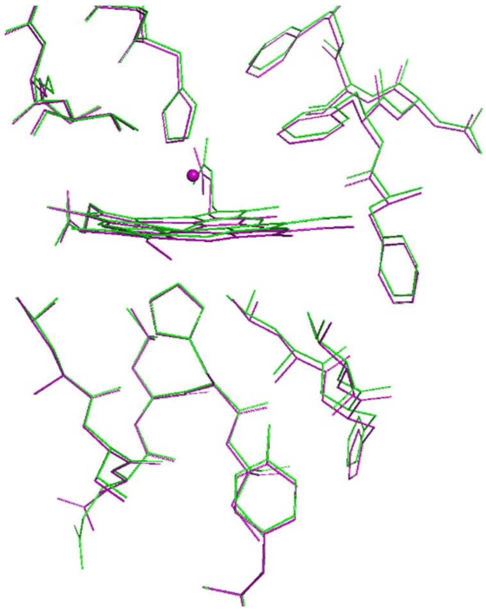

Figure S3 Comparison of the β-heme pocket of DeoxyHb (green) and MetHb (purple) in F-helix frame

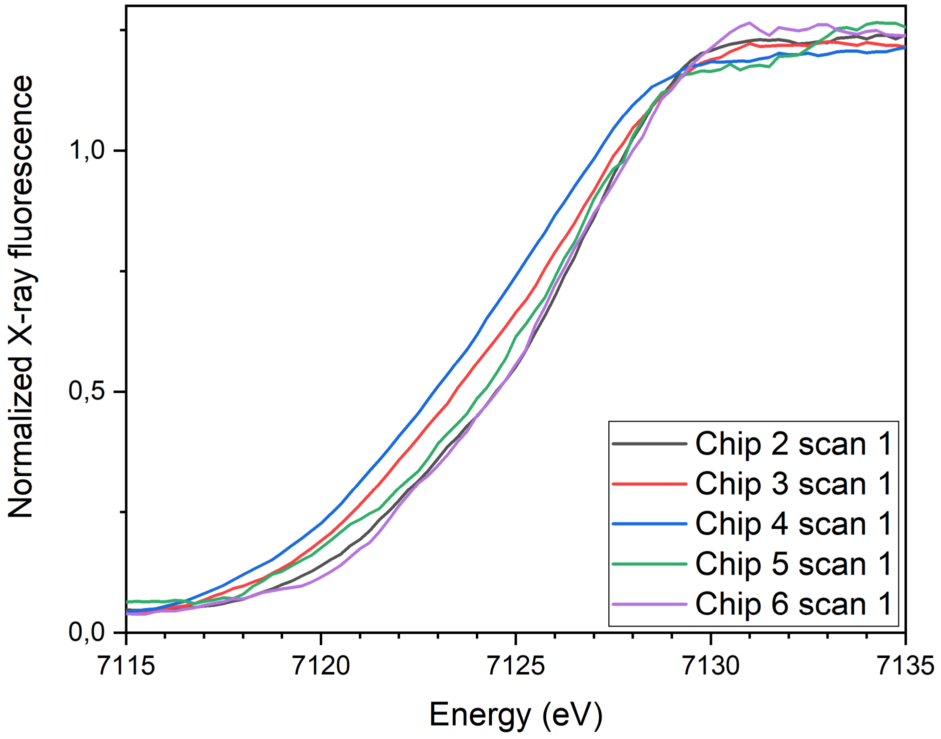

Figure S4 The first Fe K-edge XANES scan of the chip-sandwich in position 2-6 of the sample holder in Figure S2, numbered from the top left corner. Top row chip 2 (black), middle row chip 3 (red) and 4 (blue), last row chip 5 (green) and 6 (purple). T chip in position 1 (top row) was used to optimize the detector prior to the actual test.

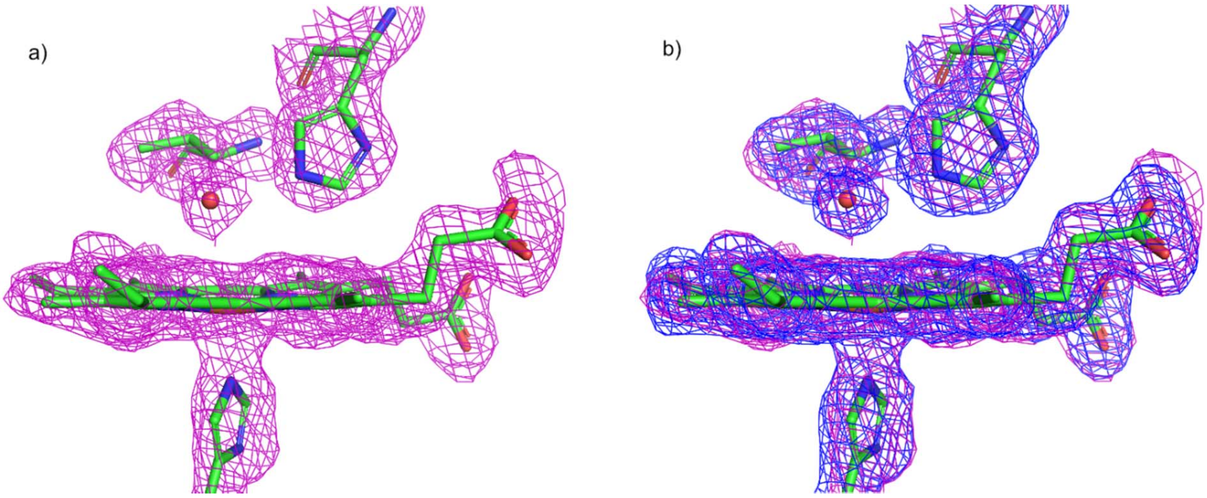

Figure S5 a) Omit electron density map (purple) for the heme pockets of DeoxyHb α subunit; b) Superimposition of the final 2mF_o–DF_c electron density map (blue) and the omit electron density map (purple) for the heme pockets of DeoxyHb α subunit. Density is contoured at 1.1σ.

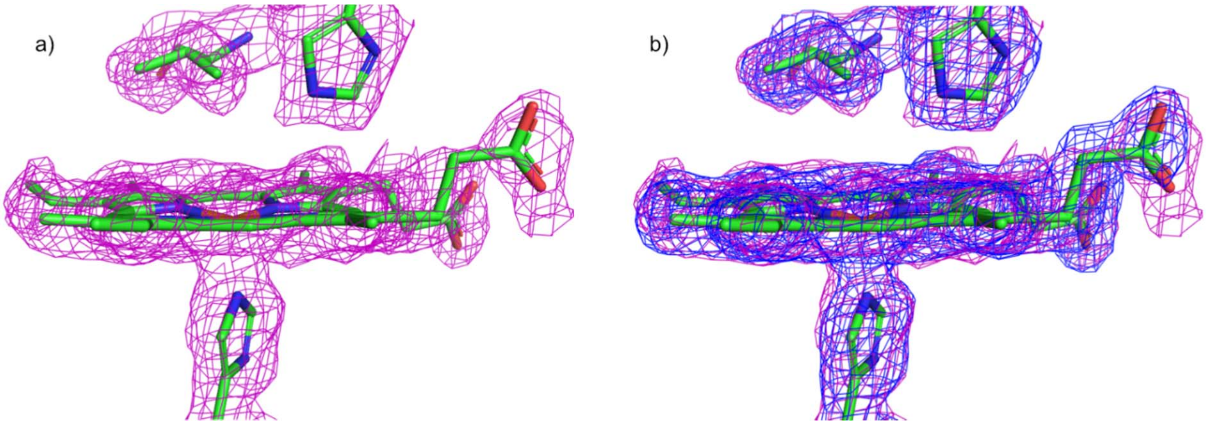

Figure S6 a) Omit electron density map (purple) for the heme pockets of DeoxyHb β subunit; b) Superimposition of the final $2mF_o–DF_c$ electron density map (blue) and the omit electron density map (purple) for the heme pockets of DeoxyHb β subunit. Density is contoured at 1.1σ.

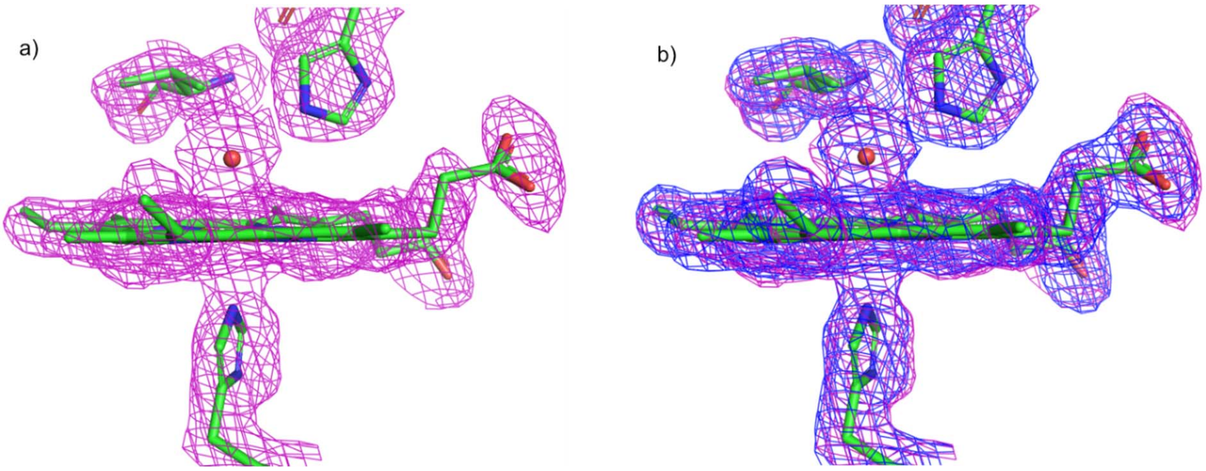

Figure S7 a) Omit electron density map (purple) for the heme pockets of MetHb α subunit; b) superimposition of the final $2mF_o–DF_c$ electron density map (blue) and the omit electron density map (purple) for the heme pockets of MetHb α subunit. Density is contoured at 1.1σ.

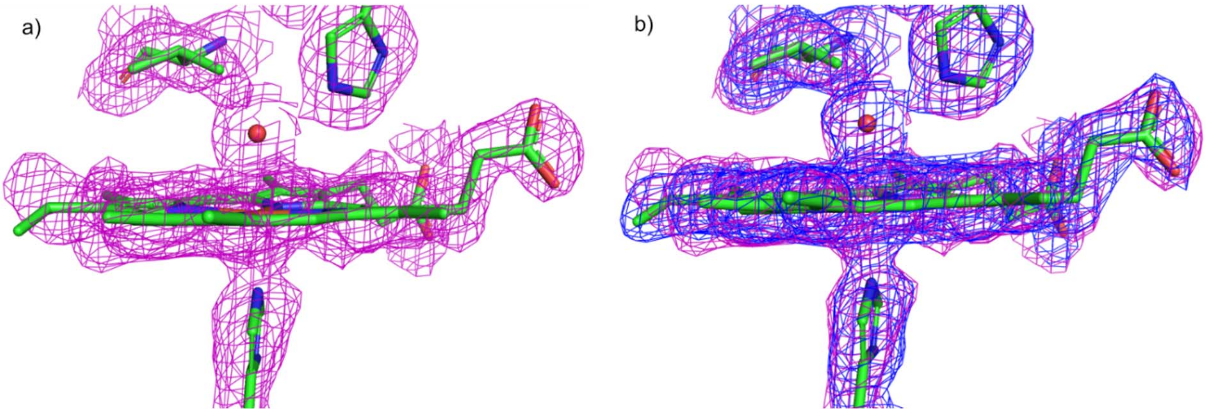

Figure S8 a) Omit electron density map (purple) for the heme pockets of MetHb β subunit; b) superimposition of the final $2mF_o–DF_c$ electron density map (blue) and the omit electron density map (purple) for the heme pockets of MetHb β subunit. Density is contoured at 1.1σ.

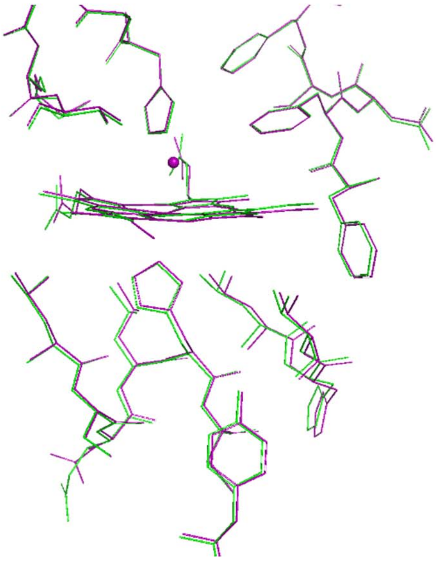

Figure S9 Comparison of β-heme pocket of DeoxyHb (green) and MetHb (purple) in BGH frame

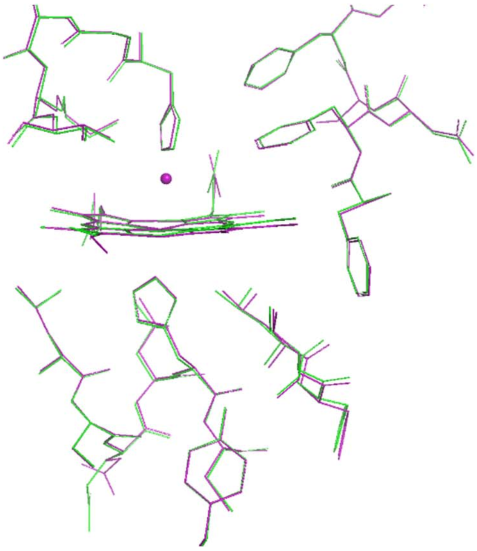

Figure S10 Comparison of β-heme pocket of DeoxyHb (green) and MetHb (purple) in heme frame

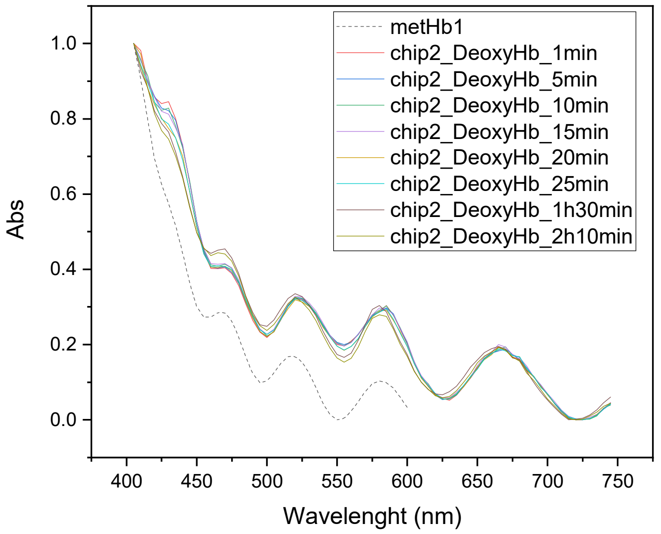

Figure S11 UV-Vis spectra of a chip-sandwich of DeoxyHb (chip_2) in the MX-sample holder (the spectra have been normalized in Origin 2018b).

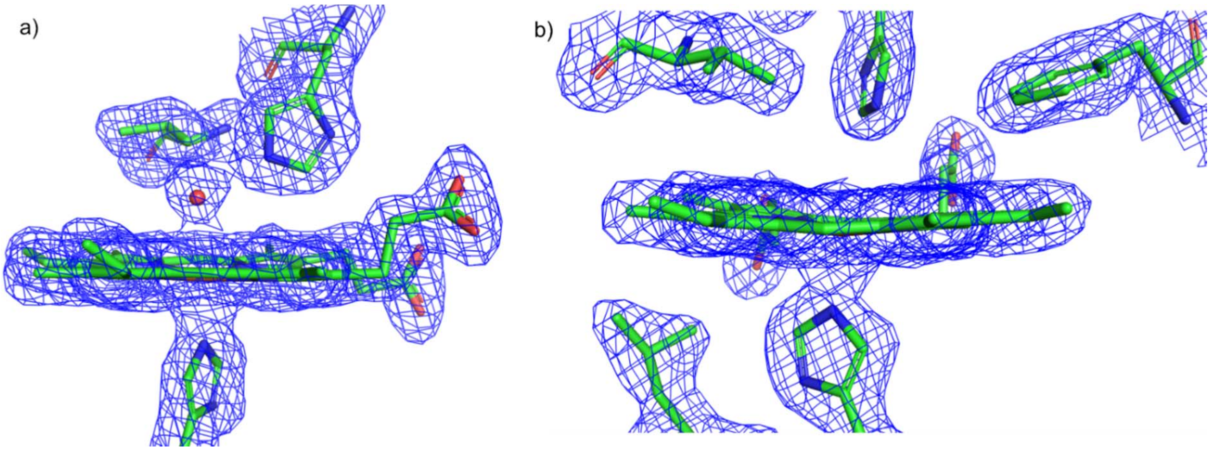

Figure S12 View of the final 2mFo–DFc electron density map for the heme pockets of chip 1 DeoxyHb, (a) the α subunit and (b) the β subunit. Density is contoured at 1.1σ. Distance between HIS58/63 and the heme iron is 4.2 Å, while distance between the water molecule and the heme iron is 3.1 Å

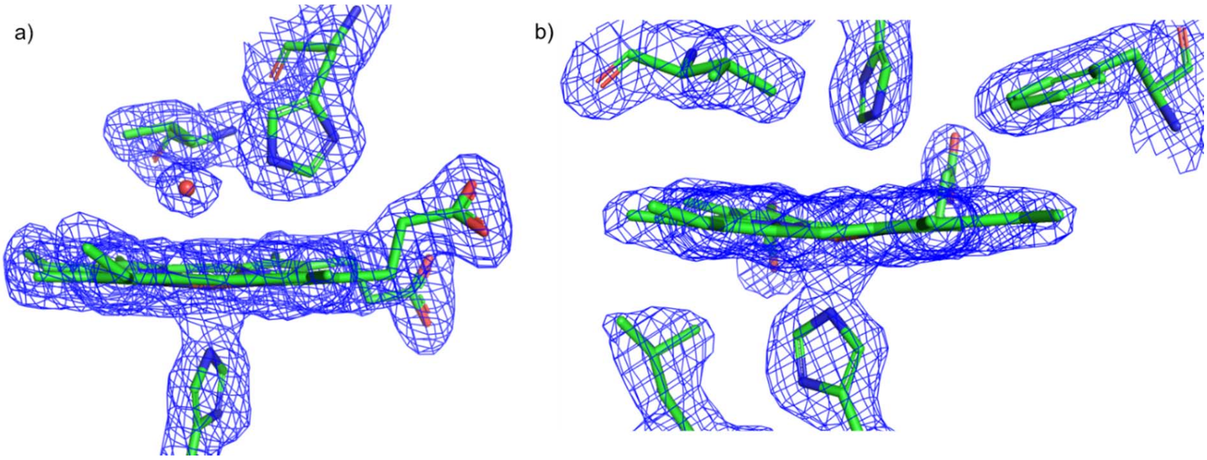

Figure S13 View of the final 2mFo–DFc electron density map for the heme pockets of chip 2 DeoxyHb, (a) the α subunit and (b) the β subunit. Density is contoured at 1.1σ. Distance between HIS58/63 and the heme iron is 4.2 Å, while distance between the water molecule and the heme iron is 3.1 Å

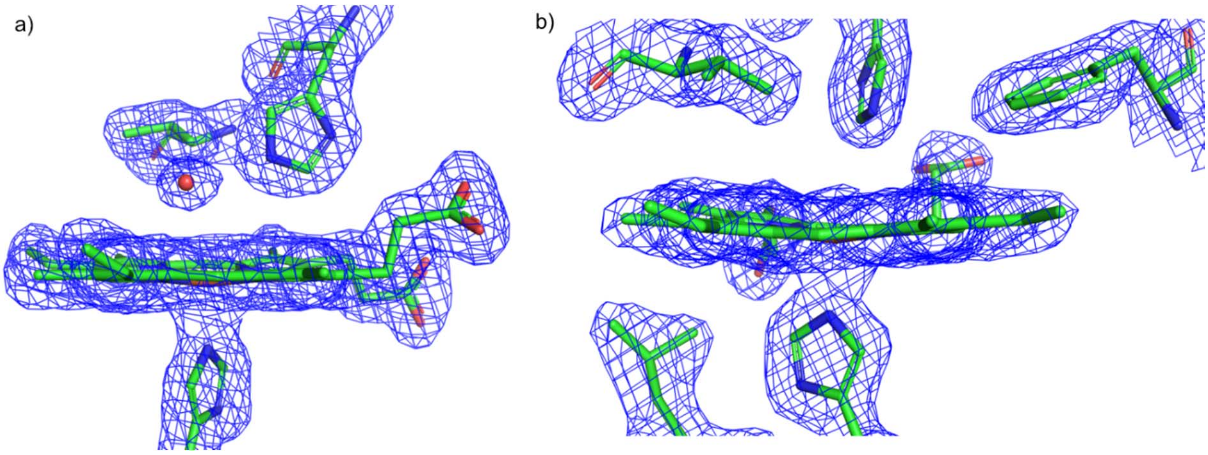

Figure S14 View of the final 2mFo–DFc electron density map for the heme pockets of chip 4 DeoxyHb, (a) the α subunit and (b) the β subunit. Density is contoured at 1.1σ. Distance between HIS58/63 and the heme iron is 4.2 Å, while distance between the water molecule and the heme iron is 3.1 Å.

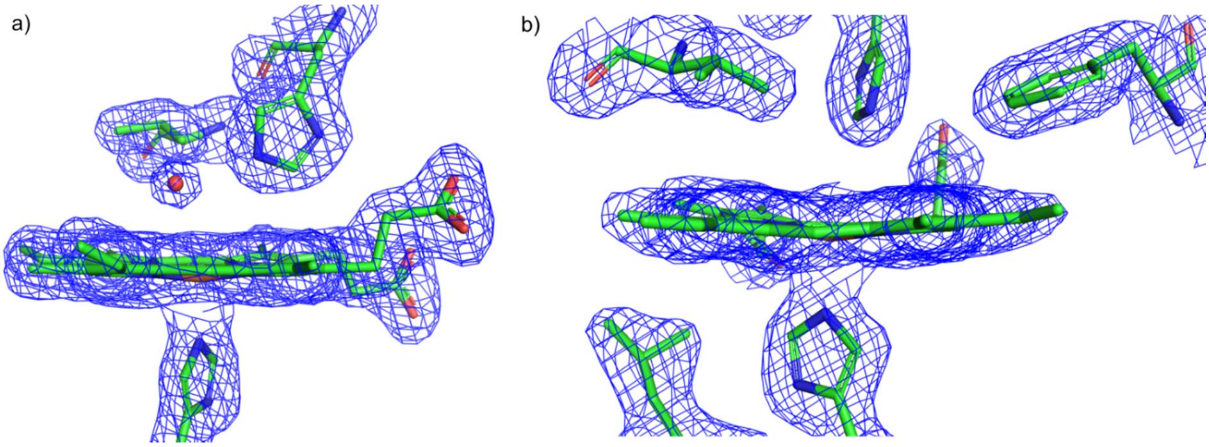

Figure S15 View of the final 2mFo–DFc electron density map for the heme pockets of chip 5 DeoxyHb, (a) the α subunit and (b) the β subunit. Density is contoured at 1.1σ. Distance between HIS58/63 and the heme iron is 4.2 Å, while distance between the water molecule and the heme iron is 3.1 Å
